# Histophatologic changes of lung in asthmatic male rats treated with hydro-alcoholic extract of *Plantago major* and theophylline

**Published:** 2013

**Authors:** Farah Farokhi, Fereshteh Khaneshi

**Affiliations:** 1 *Department of Biology, Faculty of Science, Urmia University, I. R. Iran*

**Keywords:** Asthma, Lung, Male Rats, *Plantago major*, Theophylline

## Abstract

**Objective:** *Plantago major* (*P. major*) is one of the medicinal crops in the world which has therapeutic properties for treatment of respiratory and gastrointestinal diseases. Theophylline is commonly used for the treatment of respiratory diseases. In this study, we investigated the protective effects of hydro-alcoholic extract of *P. major* on lung in asthmatic male rats.

**Materials and Methods**: 32 male adult rats were randomly divided into 4 groups: The control group (C) received normal saline; Asthma (A) group received a normal diet; Asthma group treated with Theophylline (200 mg/kg b.w.) (T); Asthma group which received* p.major* (100 mg/kg b.w.) (P). Asthma was induced by citric acid, 0.1 mg in form of spraying. The injection of* P.major* extract and theophylline was administered intraperitoneally for four weeks. At the end of the treatment, all of the rats were sacrificed and lungs were taken out, fixed, and stained with H&E, toluidine blue, and PAS, then histological studies were followed with light microscope.

**Results:** Results showed that, in asthmatic group, the mean number of mast cells was significantly increased (p<0.05). Thickness of alveolar epithelium and accumulation of glycoprotein in airways was increased. Moreover, in some of alveolar sac hemorrhaging was observed. Administration of *p.major *extract in asthmatic rats restored these changes towards normal group.

**Conclusion**: The present study revealed that *P. major* compared with theophylline, has a protective effect on lung in asthmatic rats.

## Introduction

Airways inflammation is thought to play a central role in the pathogenesis of asthma. Clinical investigations show a correlation between the presence of activated inflammatory cells (neutrophils, mast cells, and eosinophils), histological changes to pulmonary tissue, and the development of airways hyperreactivity (Wagelie steffen et al., 2006 ▶). Although there is no cure for asthma, there are ways to control and prevent symptoms. Drugs used in asthma have been divided into two categories: drugs that cause dilated respiratory tract and preventive medications that suppresses inflammation and prevent symptoms of respiratory tract diseases. Asthma symptoms are removed with specific mechanisms of enzyme function in the airway cells (Boskabady et al., 2002 ▶). One of the anti-asthma drugs is theophylline which belongs to xeanthin family that causes dilation of the smooth muscles and eliminates sudden obstruction (Sohrabi et al., 2009 ▶). Traditionally, using medicinal plants for curing of respiratory diseases has been considered many years ago (Rahimian, 2002 ▶). Researchers have been reported that ginger plants could be effective in treating asthma. *Plantain *is found all over the world and is one of the most abundant accessible medicinal herbs (Green, 2000 ▶). It contains many bioactive compounds including allantoin, aucubin, ursolic acid, flavonoids, and asperuloside. Scientific studies have shown that plantain extract has a wide range of biological effects including "wound healing activity, anti-inflammatory, analgesic, antioxidant, weak antibiotic, immunomodulating, and anti-ulcerogenic activity" (Samuelsen, 2000 ▶). 

The root of *plantain* was traditionally used to treat wounds, as well as fever and respiratory infections. This plant consists of phenol combinations and has antioxidant property (Mehrabian et al., 2009 ▶). Its leaf (duo to consist of Tanen and Mucilage) is beneficial in the treatment of the inflammation of the upper portion of respiratory system. (Razavi et al, 2009 ▶).* Plantago major* *(P. major)* with specific polysaccharides improves wound. A research has shown that *P. major* can be effective in the treatment of chronic bronchitis (Tilford et al., 1997 ▶). This research revealed that* Plantago lanceolata* has antitussive effects in Guinea pigs (Boskabady et al., 2006 ▶). In this research, the antiasthmatic effect of *P. major* was evaluated.

## Materials and Methods

**Preparation of extracts**

Fresh, green *P. major *plants were collected from Urmia highland in May 2011 and authenticated by a professor from the Department of Biology at Urmia University with herbarium number of 3996. The leaves of samples were dried in shadow at room temperature for seven days. Collected samples were ground by an electrical mill. One hundred grams of powder samples were added to 1000 ml ethanol 96%, then after 24 h, the solution was filtered. In the second step, ethanol 70% was added to the remained dry materials. After 24 h, the solution was filtered and then evaporated repeatedly to half of the first volume by rotary evaporator in 50 ºC and 70 rpm. Concentrated extracts were dried on water bath at 40 ºC temperature to prepare injected extract. This powder was solved in specific volume of normal saline (Boskabady et al., 2006 ▶). 

**Animal treatment**

The current study was conducted on 32 healthy adult male Wistar rats weighing 200±20 g. Animals were kept in standard conditions with constant 12h light/dark cycle at temperature of 22±5 ºC. The rats were fed with standard diet. Rats were purchased from Pasteur Institute, Tehran, Iran. The animals were handled in accordance with the Institutional Guidelines for the Care and Use of Animals for Experimental Purpose. The animals were acclimatized to the animal room condition for two weeks prior to the experiment. These rats were divided into equal four groups with 8 rats per group as follows: The first control group received normal saline. The experimental groups were asthmatic rats. The second group (A): untreated asthma, received normal diet. The third asthmatic group was injected Theophylline 200 mg/kg b.w. intraperitoneally (T). The fourth asthmatic group received intraperitoneally hydro-alcoholic extract of *p. major* which was dissolved in normal saline with dosage of 100 mg/kg b.w. (P)**.**Treatment periods were 4 weeks and drugs were administered intraperitoneally with insulin needle.

**Induction of asthma in rats**

Asthma was induced by citric acid 0.1 mg in form of spraying for seven minutes along fifteen days. Asthma symptoms including wheezing (a whistling sound when breathe), chest tightness, shortness of breath, and coughing were observed (Bartlett et al., 2008 ▶).

**Tissue preparation**

At the end of the experiment, the rats were anesthetized by chloroform and their lungs were taken out and fixed in 10% natural buffer formalin. After tissue processing, the samples were blocked in paraffin blockers and then stained with H&E, toluidine blue, and PAS. Histopathological studies were followed by light microscope. Mast cells were counted with netted lenses in the unit area (mm²). 

**Statistical analysis**

All data were expressed as mean±SEM. One-way ANOVA followed by Tukey´s post hoc test for comparisons were used for statistical evaluation. Statistical significance was accepted at p<0.05. The graphs were drawn by Excell software, 2007.

## Results

**Figure 1:**
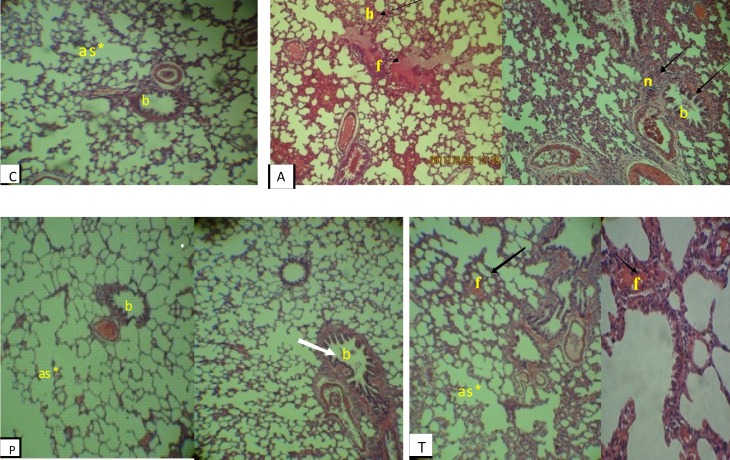
Cross sections of lung in experimental groups stained with H&E, 40×. C: Normal control; A: Asthma group; P: *Plantago grou*p; T: Theophylline group; Lymphoid nodules (n); alveolar sac (as); epithelium (ep); hemorrhage (h); replete blood (f); bronchiole (b).

Histopathological study of lung tissue in healthy normal control group revealed the alveolar sac and bronchioles with normal epithelium. In asthmatic group, the accumulation of inflammatory cells and lymphoid nodules around the interaalveolar septum was seen. Moreover, thickness of epithelium in the alveoli was increased with multiple centers of hemorrhage in some of alveoli and replete with blood in the connective tissue between alveoli were observed. In the group treated with the extract of *plantago*, the thickness of epithelium was decreased and improved, meanwhile presence of lymphoid cells was less and scattered. In this group, bronchioles and alveolar sacs were similar to the control group. In the group treated with theophylline compared with asthma group, the thickness of epithelium was reduced, while in some of the alveoli, the thickness of epithelium was increased and replete with blood was observed (Figure 1).

**Figure 2:**
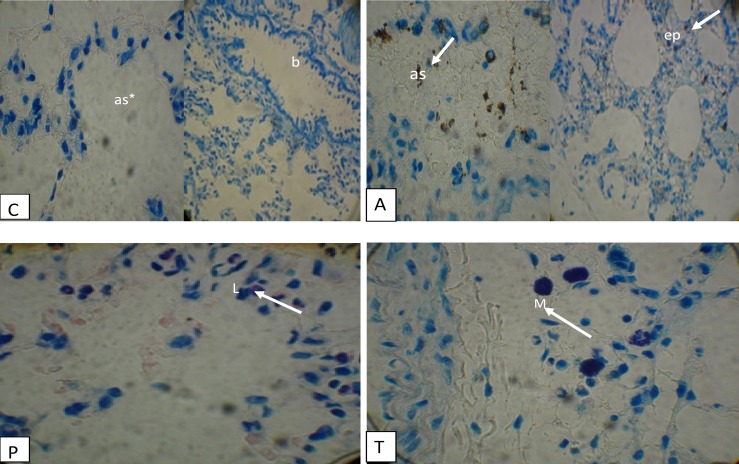
Cross sections of lung in experimental groups stained with toluidine blue, 400×. C: Normal control; A: Asthma group; P: *Plantago group;* T: Theophylline group; epithelium (ep); mast cell (M); alveolar sac (as); bronchiole (b); lymphocyte (L).

Histopathological study with toluidine blue staining revealed the reduction of the size and number of mast cells in the control group. Moreover, alveolar sac and bronchioles with normal height epithelium were seen. In asthmatic group, thickness of alveolar epithelium, the size, and number of mast cells was significantly increased (p<0.05). Furthermore, multiple centers of hemorrhage in some of alveolar sacs were observed with presence of hemosiderin pigments. In the P group which was treated with extract of *p. major*, the thickness of epithelium was recovered, the size and the number of lymphoid cells were less and scattered. In the theophylline group compared with the asthma group, the thickness of epithelium was reduced, but number and size of mast cells was similar to the asthma group (Figure 2).

**Figure 3:**
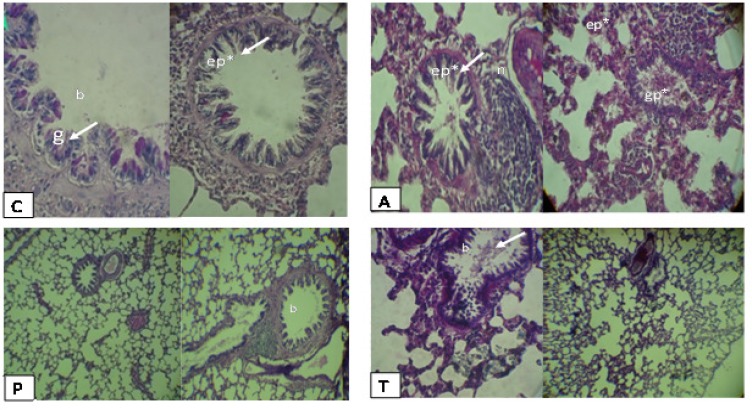
Cross sections of lung in experimental groups, stained with PAS, 100×. C: Normal control; A: Asthma group; P: *Plantago group*; T: Theophylline group; epithelium (ep); glycoprotein (gp); bronchiole (b); lymphatic nodule (n), goblet cell (g). This figure is showing increasing of glycoprotein compounds in bronchial of asthmatic and theophylline groups, while reduction of these symptoms in control and *plantago groups*

Histopathological study in the asthma group with PAS staining revealed that the glycoprotein compounds accumulates in airways and all of goblet cells in bronchioles were emptied and height of bronchiolar epithelium was increased. Moreover, thickness of epithelium in the alveoli was increased and a lot of large nodules around bronchioles were observed. While in the* plantago* groups, the bronchioles and air sacs were similar to the control group, meanwhile presence of lymphoid cells was less and scattered. In the group treated with theophylline not only all of goblet cells in bronchioles were evacuated compared with the control group, but also presence of glycoprotein compounds in airways was conspicuous. The thickness of epithelium was reduced, but in some alveoli it was similar to asthma group (Figure 3).

**Figure 4:**
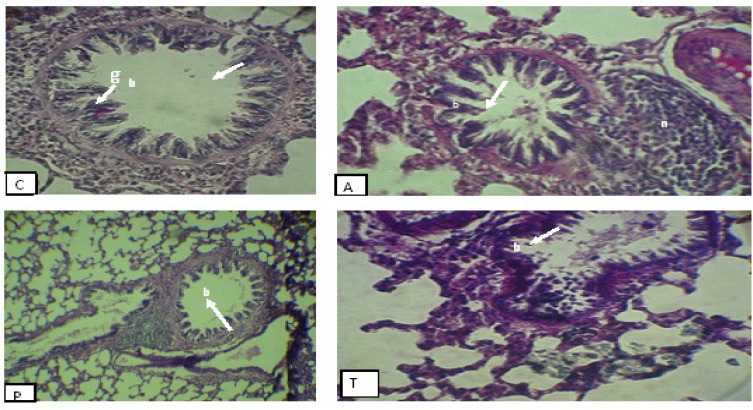
Cross section of bronchial in experimental groups with PAS staining, 100×: C: Normal control; A: Asthma group; P*: Plantago gro*up; T: Theophylline group; Lymphoid nodules (n); goblet cell (g); bronchiole (b). As shown in this figure, in the control group (b) bronchial was empty and without any secretion compounds, but a lot of goblet cells (g) with normal epithelium were observed. In the asthmatic group, bronchioles were filled with glycoprotein compounds and height of epithelium was increased. Furthermore, a few lymphatic nodules (n) around bronchioles were observed. In this group, most of goblet cells were emptied. In the theophylline group, height of epithelium and glycoprotein compounds was reduced, meanwhile some normal goblet cells were observed. In the *plantago *group, similar to control group, bronchial was empty and goblet cells with normal epithelium were observed

**Figure5:**
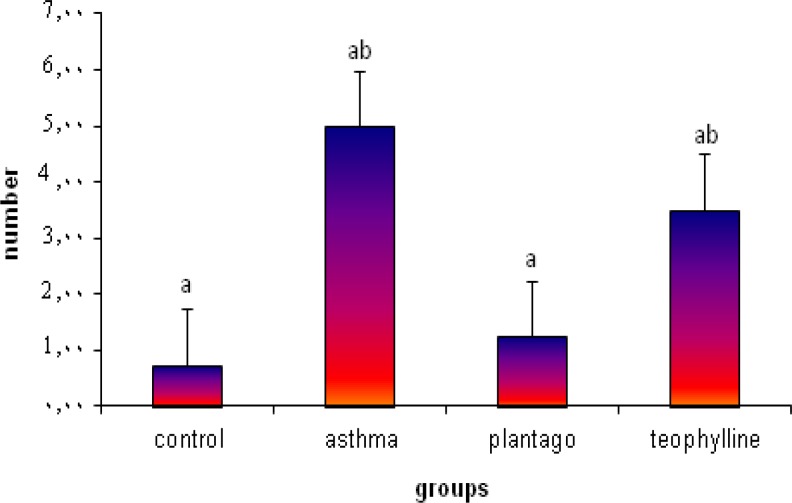
The mean number of mast cells in mm² in the experimental groups. Values are statistically significant at p<0.05 in differ characters.

As shown in this figure, the mean number of mast cells in mm² in asthmatic group was significantly increased in comparison with the control and plantago groups (p<0.05). In the theophylline group, the mean number of mast cells in mm² was significantly increased (p<0.05) in comparison with the control and plantago groups, while plantago group was similar to the control group (p>0.05). Furthermore, there was no significant decrease in theophylline group compared with the asthmatic group (p>0.05).

## Discussion

It is important that mouse models are valid and reflect clinical asthma as closely as possible.Animal models have been used to elucidate asthma and to identify and evaluate novel therapeutic targets.

Stimulating factors for asthma is related to the accumulation of inflammatory cells in airways (Makhlogh et al., 2006 ▶). It has been proved that airwayʼs neutrophils are increased in asthma (Zhou et al., 2001 ▶). IL-17 may be involved in the infiltration of neutrophils into the lung tissue (Sergejeva et al., 2005 ▶). Neutrophils secrete elastase, which is a neutrophil serine protease that causes degeneration of lung elastin. The production of reactive oxygen is activated and airway inflammation is increased (Khosrou et al., 2006 ▶). IL-17 is one of the cytokins that may increase inflammation by attracting neutrophils to the peripheral tissues (Hellings et al., 2003 ▶; Molet et al., 2001 ▶). Neutrophils secrete metalloproteinase (MMPs) which have destructive effects on tissue. Some researchers have proved that as the number of mast cells in asthma is increased, the density of free radicals after immunological stimulation increases (poli et al., 2004 ▶). Furthermore, there is little or no recruitment of mast cells into the airway wall or epithelium (Boyce and Austen, 2005 ▶) which may reflect the paucity of mast cells in the airways of mice (Kumar and Foster, 2002 ▶). In the present study, it was observed that in asthmatic group, number of lymphoid and mast cells increased. Moreover, the glycoprotein compounds accumulate in airways and all of goblet cells in bronchioles were emptied. In order to compensate for these changes, the thickness of epithelium in the alveoli was increased and some large nodules around bronchioles were observed. Therefore, multiple centers of replete with blood in the connective tissue between alveoli and hemorrhage in some alveolar sacs were observed. Hemosiderin particles were scattered in alveoli, thus making the breathing difficult. 

Theophylline has been a popular medication for asthma for over 50 years. It causes dilation of the smooth muscles and eliminates sudden obstruction (Sohrabi et al., 2009 ▶). It inhibits phosphodiesterase enzyme, which is responsible for cAMP destruction. With increase of cAMP, respiratory tract is dilated and asthma symptoms usually go away (Boskabady et al., 2002 ▶). Theophylline down-regulates the function of inflammatory and immune cells in vitro and in vivo in animals with airway inflammation (Scordamaglia et al., 1988 ▶). In patients with allergic asthma, it attenuates the late-phase increase in airway obstruction and airway responsiveness to histamine and decreases allergen-induced migration of activated eosinophils into the bronchial mucosa (Sullivan et al., 1994 ▶). The bronchoprotection may be effected by direct inhibition of smooth-muscle contraction. In contrast, attenuation of the early response to allergens or exercise by theophylline may involve inhibition of the release of leukotrienes from the airways, attenuation of the effects of leukotriene D4 at its receptor, or blocking of adenosine-induced enhancement of mediator release from mast cells (Welton et al., 1980 ▶).

Sanati and colleagues in 2009 reported the effect of theophylline in patients with syndrome X (chest). Theophylline could reduce the chest pain with non-specific inhibitor adenosine receptors. Pour Abuli and colleagues in 2008 revealed that inhibition of adenosine receptors by theophylline caused the reduction of inflammatory edema, vascular permeability, and vasodilatation. In the present research, it was revealed that in the group treated with theophylline compared with the asthma group, the thickness of some alveolar epithelium was reduced and the accumulation of inflammatory cells and lymphoid nodules around the septum was smaller than the asthma group. Moreover, presence of glycoprotein compounds in airways was reduced, and the height of bronchial epithelium was recovered. However, size of mast cells and macrophages was increased.

Modern medical research is proving to uphold many of the historical uses of *plantain* (Mukhtar et al., 2008 ▶) especially as a wound healer and as a treatment for lung conditions such as bronchitis and asthma (Galvez et al., 2003 ▶). Medicinally,platanin is used for mucous membrane irritation associated with upper respiratory tract. Two Bulgarian clinical trials have suggested that *plantain *may be effective in the treatment of chronic bronchitis (Tilford, 1997 ▶). *Plantain* has been used medicinally for centuries. It was considered that historically it has been recommended as a treatment for just about everything, such as ulcers, ringworm, jaundice, epilepsy, liver obstructions, and hemorrhoids! *Plantain* was so commonly known that it is even found referenced in works by both Chaucer and Shakespeare (Tilford et al., 1998). Previous study demonstrated that *plantago lanceolata* in four weeks in Guinea pigs has sedative and anti-cough properties. (Boskabady et al., 2006 ▶). The leaf of *plantago* prevents inflammation of the mouth (stomatitis) in chemotherapy (Pour Ismail et al., 2003 ▶).

*P. major* is one of the most abundant and widely distributed medicinal crops in the world. The active chemical constituents are aucubin (an anti-microbial agent), allantoin which stimulates cellular growth and tissue regeneration (Velasco-Lezama et al., 2006 ▶), and mucilage which reduces pain and discomfort (Duke et al., 2001 ▶). Together, these constituents are thought to give *plantain* mild anti-inflammatory, antimicrobial, antihemorrhagic, and expectorant actions. In this research, in the group treated with the extract of *plantago* due to allantoin constituents, goblet cells in bronchioles were recovered and regenerated. Furthermore, due to mucilage compounds in *plantago*, the rate of lymphoid cells and nodules were small and decreased. Moreover, the thickness of epithelium was decreased and improved. The bronchioles and air sacs were observed to be similar to the control group.

In summary, the results of this research revealed that *p. major* compared with theophylline, prevent histopathological changes of lung in asthmatic rats. The effect can be due to Tanen and Mucilage. Anti-inflammatory, analgesic, antioxidant, immunomodulating and anti-ulcerogenic activity of this plant has a protective effect on lung in asthmatic rats. Therefore, it is beneficial in the treatment of the inflammation of the upper part of respiratory system.
